# A-Type K_V_ Channels in Dorsal Root Ganglion Neurons: Diversity, Function, and Dysfunction

**DOI:** 10.3389/fnmol.2018.00253

**Published:** 2018-08-06

**Authors:** Benjamin M. Zemel, David M. Ritter, Manuel Covarrubias, Tanziyah Muqeem

**Affiliations:** ^1^Vollum Institute, Oregon Health and Science University, Portland, OR, United States; ^2^Division of Neurology, Cincinnati Children's Hospital Medical Center, Cincinnati, OH, United States; ^3^Department of Neuroscience, Vickie and Jack Farber Institute for Neuroscience, Sidney Kimmel Medical College and Jefferson College of Life Sciences at Thomas Jefferson University, Philadelphia, PA, United States

**Keywords:** Kv channel, A-type, dorsal root ganglion, pain, Kv1.4, Kv3.4, Kv4

## Abstract

A-type voltage-gated potassium (Kv) channels are major regulators of neuronal excitability that have been mainly characterized in the central nervous system. By contrast, there is a paucity of knowledge about the molecular physiology of these Kv channels in the peripheral nervous system, including highly specialized and heterogenous dorsal root ganglion (DRG) neurons. Although all A-type Kv channels display pore-forming subunits with similar structural properties and fast inactivation, their voltage-, and time-dependent properties and modulation are significantly different. These differences ultimately determine distinct physiological roles of diverse A-type Kv channels, and how their dysfunction might contribute to neurological disorders. The importance of A-type Kv channels in DRG neurons is highlighted by recent studies that have linked their dysfunction to persistent pain sensitization. Here, we review the molecular neurophysiology of A-type Kv channels with an emphasis on those that have been identified and investigated in DRG nociceptors (Kv1.4, Kv3.4, and Kv4s). Also, we discuss evidence implicating these Kv channels in neuropathic pain resulting from injury, and present a perspective of outstanding challenges that must be tackled in order to discover novel treatments for intractable pain disorders.

## Introduction

**Figure 1:**
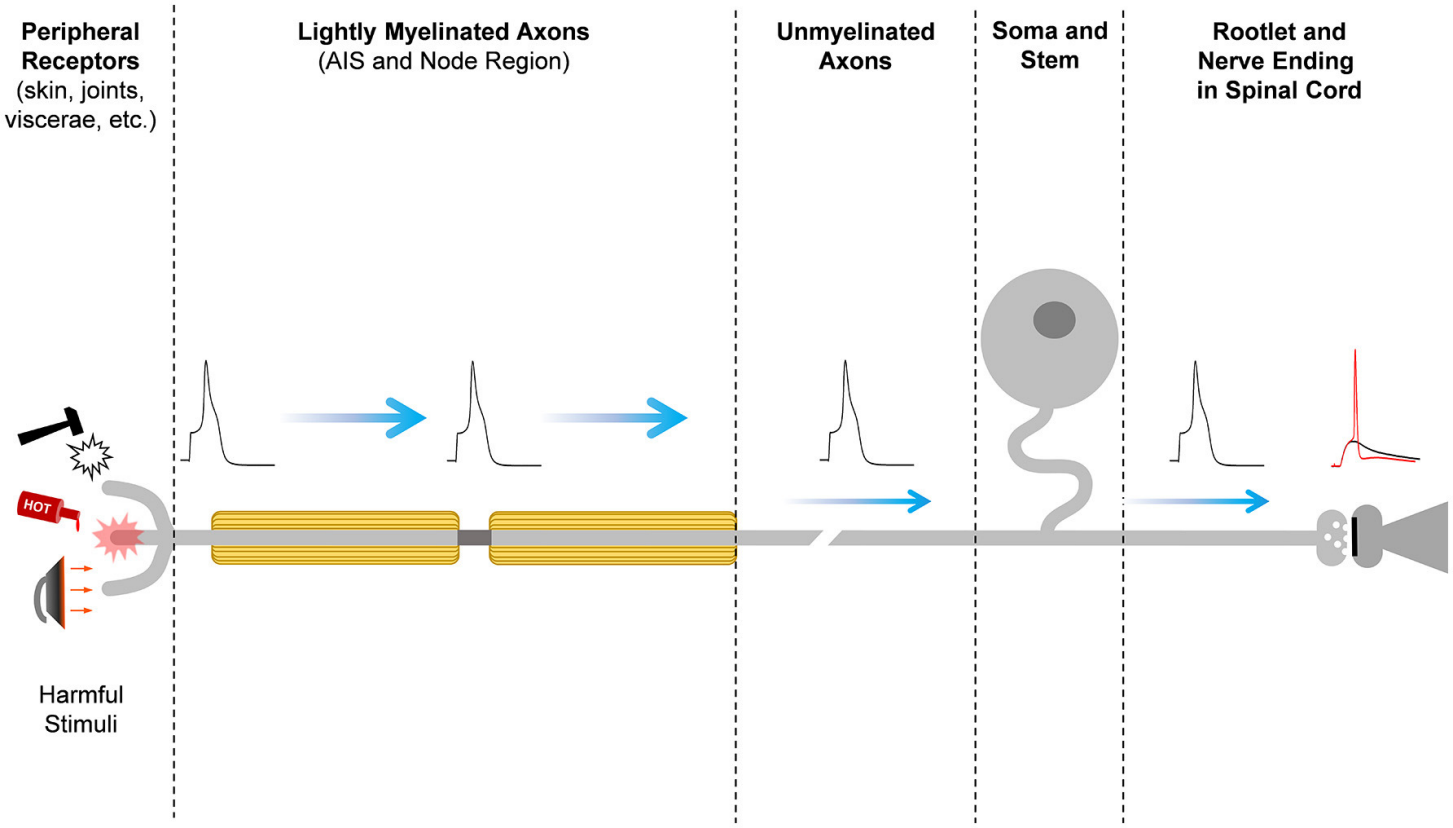
Pain signal propagation in stereotypical nociceptive neurons of the mammalian DRG. The initiation, propagation, firing frequency, and shape of the action potential that carries nociceptive information involves a large number of diverse ion channels with heterogeneous subcellular distributions along the primary sensory neurons in the DRG. Mechanical, thermal, chemical, and inflammatory stimuli activate receptor ion channels in the periphery, giving rise to a depolarizing receptor potential that might be large enough to initiate a nerve impulse at the action potential initiation site (AIS). Firing of the action potential and the properties of the action potential waveform depend on the activities of an ensemble of voltage-gated ion channels and Ca^++^-dependent K^+^ channels. The action potential of nociceptors propagates centrally along lightly myelinated A-delta fibers and unmyelinated C-fibers until it reaches the nerve ending in the superficial dorsal horn of the spinal cord, where it evokes Ca^++^-dependent vesicular release of the excitatory neurotransmitter glutamate. At the glutamatergic nerve ending, certain Kv channels can shape the action potential to regulate voltage-dependent Ca^++^ influx into the nerve ending and, consequently, nociceptive synaptic transmission. Glutamate excites the secondary sensory neuron to relay the nociceptive information that eventually reaches pain perception centers in the brain.

Inactivating voltage-gated K^+^ (Kv) currents were first characterized in neurons from the marine gastropod *Onichidium verruculatum* and were subsequently described as “A-type” (Hagiwara et al., 1961; Nakajima, 1966; Connor and Stevens, 1971a,b; Neher, 1971). Although distinct voltage-dependencies of inactivation and sensitivities to K^+^ channel antagonists allowed the functional dissection of A-type Kv currents, the molecular correlates remained unknown for many years. The cloning of the gene encoding the *Drosophila* Shaker channel opened the door to the discovery of homologous Kv channel genes from vertebrates and a better understanding of the diversity, structure, function, and modulation of specific A-type Kv channels (Papazian et al., 1987; Rudy, 1988; Stühmer et al., 1989; Pak et al., 1991; Salkoff et al., 1992). Mammalian A-type Kv channels include: Kv1.4 (KCNA4), Kv3.3 (KCNC3), Kv3.4 (KCNC4), Kv4.1 (KCND1), Kv4.2 (KCND2), and Kv4.3 (KCND3) (Stühmer et al., 1989; Baldwin et al., 1991; Pak et al., 1991; Rudy et al., 1991; Schröter et al., 1991; Vega-Saenz de Miera et al., 1992; Serôdio et al., 1996). Although the discovery of these genes was transformational, the identification and reconstitution of the native channels underlying the corresponding diversity of A-type Kv currents in the nervous system has been challenging. The discovery of Kv channel accessory subunits has helped determine the function, modulation and diversity of Kv channels in their native neuronal environment (Covarrubias et al., 2008; Maffie and Rudy, 2008; Marionneau et al., 2009; Kanda and Abbott, 2012; Weingarth et al., 2013; Jerng and Pfaffinger, 2014). Knockout animals and knockdown techniques are also helping dissect the molecular correlates and function of fast inactivating potassium currents in the nervous system (Malin and Nerbonne, 2000, 2001; Akemann and Knöpfel, 2006; Hu et al., 2006; Hurlock et al., 2008; Zagha et al., 2008; Norris et al., 2010; Carrasquillo et al., 2012; Ritter et al., 2012; Rowan et al., 2016; Hermanstyne et al., 2017; Kaczmarek and Zhang, 2017). The rat dorsal root ganglion (DRG) mainly expresses Kv1.4, Kv3.4, Kv4.1, and Kv4.3, which will be the primary focus of this review (Figure 1, Table 1). Although the membrane currents produced by these A-type Kv channels exhibit similar fast inactivating profiles, their subcellular distribution, biophysical properties, and mechanisms of inactivation and modulation differ greatly (Table 1). For instance, whereas Kv1.4 and Kv3.4 channels are generally found in axons and nerve endings, Kv4 channels are generally somatodendritic in the central nervous system (Sheng et al., 1992; Trimmer and Rhodes, 2004; Strassle et al., 2005; Lai and Jan, 2006; Kim and Hoffman, 2008; Clark et al., 2009; Huang et al., 2017). Therefore, A-type Kv channels play distinct roles along different subcellular compartments of diverse neuronal subtypes. Determining these roles in heterogeneous and highly specialized DRG neurons and the pathophysiological implications are topical subjects of active investigation. Multiple reviews have been recently published on the roles of diverse DRG ion channels on pain signaling under physiological and pathological conditions (Rasband et al., 2001; Dib-Hajj et al., 2009; Cregg et al., 2010; Dubin and Patapoutian, 2010; Julius, 2013; Wemmie et al., 2013; Tsantoulas and McMahon, 2014; DiFrancesco and DiFrancesco, 2015; Bernier et al., 2017; Queme et al., 2017). However, to the best of our knowledge, no specific reviews have been published on the function, dysfunction and diversity of A-type Kv channels in the DRG, which are likely to play specialized critical roles in different compartments of primary sensory neurons. This article attempts to fill this gap by reviewing original discoveries in this area including recent studies demonstrating the physiological and molecular properties of A-type Kv channels in the pain pathway and how their dysfunction might contribute to pathological pain states. Ultimately, this knowledge would stimulate further work to better understand these ion channels and help identify viable therapeutic interventions to treat pain disorders.

**Table 1:** Biophysical properties of A-type Kv channels expressed in native and heterologous expression systems.

**Kv channel**	**Expression ^a^ system**	**γ pS**	***V*_1/2_ Activation mV**	***Z* Act. e_0_**	***V*_1/2_ Inactivation mV**	***Z* Inact. e_0_**	**Tau^b^ deactivation ms (Voltage)**	**Tau^c^ inactivation ms (Voltage)**	**Tau^d^ recovery ms (Voltage)**	**References**
Kv1.4	Native/DRGN	−	−9.2	1.7	−57	2.9	2.5 (−100)	8.4 (+30)	−	Gold et al., 1996
Kv1.4	*X*. oocyte	9.3	−21.7	1.5	−74	2	1 (−140)	50 (+20)	1040 (−80)	Stühmer et al., 1989; Tseng-Crank et al., 1993; Jerng et al., 1999
Kv1.4/Kvβ1.1	*X*. oocyte	−	−	−	−54	7.7	−	4 (+50)	2000 (−100)	Rettig et al., 1994
Kv1.4	HEK−293	−	−48	4.3	−65	8.8	−	49 (+40)	−	Kupper, 1998
Kv3.4	Native/DRGN	15.5	21.6	1	−25	2.3	1	15 (+60)	1800 (−100)	Ritter et al., 2012
Kv3.4	*X*. oocyte	14	23	1.9	−25	2.6	−	18.9 (+50)	1200 (−100)	Schröter et al., 1991; Beck et al., 1998
Kv4.1,4.3	Native/DRGN	−	−33	1.7	−86	3.6	−	190 (+60)	60 (−120)	Phuket and Covarrubias, 2009
Kv4.1^e^	*X*. oocyte	5.1	−4	0.9	−69	5	2.4 (−140)	150 (+60)	171 (−100)	Beck and Covarrubias, 2001; Beck et al., 2002
Kv4.1/KChIP1	*X*. oocyte	5.3	−9.4	1.1	−58	6.7	1 (−140)	77 (+60)	43 (−100)	Beck et al., 2002
Kv4.2	Native/CGN	7.8	−8.5	0.82	−77.5	2.2	−	28 (−25)	11 (−130)	Fineberg et al., 2012
Kv4.2	tsA−201	4.1	−13.2	1.1	−81	4.5	−	29 (+60)	186 (−115)	Dougherty and Covarrubias, 2006; Amarillo et al., 2008
Kv4.2/KChIP1	CHO/tsA-201	−	−8	1.1	−67	5	−	72 (−20)	96 (−140)	Amarillo et al., 2008; Maffie and Rudy, 2008
Kv4.2/DPP6-s	tsA-201	7.7	−28	0.94	−87.5	5.2	−	10 (+60)	116 (−115)	Dougherty and Covarrubias, 2006; Amarillo et al., 2008; Fineberg et al., 2012
Kv4.2/KChIP1/DPP6 -s	tsA-201	7.8	−7.3	0.78	−89.3	3.6	−	18 (−25)	45 (−140)	Amarillo et al., 2008; Fineberg et al., 2012
Kv4.2/KChIP1/DPP10a	tsA-201	−	9.4	0.6	−71	4.5	−	18 (+60)	−	Fineberg et al., 2012
Kv4.3	*X*. oocyte	4.4	1.6	1	−62	6.7	3 (−140)	86 (+60)	120 (−100)	Beck et al., 2002; Holmqvist et al., 2002; Kaulin et al., 2008, 2009
Kv4.3/KChIP1	*X*. oocyte	−	−14	1.2	−68	5.7	1 (−140)	60 (−20)	25 (−100)	Beck et al., 2002; Kaulin et al., 2008
Kv4.3/DPP6-s^f^	*X*. oocyte	6.9	−32	1.3	−81	5.7	1 (−140)	14 (+60)	70 (−100)	Kaulin et al., 2009

a *DRGN and CGN refer to dorsal root ganglion neuron and cerebellar granule neuron, respectively. Data from a putative Kv1.4 channel expressed in DRGN are mainly from cells with a diameter >25 μm. Kv3.4 and Kv4.1,4.3 DRG data are mainly from cells with diameters ≤ 20 μm and between 25 – 30 μm, respectively. Heterologous expression includes Xenopus oocytes (X. oocyte) and mammalian cell lines (HEK293, tsA-201 and CHO)*.

b *Time constants of deactivation are reported at strongly hyperpolarized membrane potentials, where channel closing is expected to dominate gating kinetics*.

c *Time constants of inactivation are reported at the membrane potential that yielded the shortest value. These values were estimated from a single exponential fit to the macroscopic decay of the current, or from the weighted sum of the derived time constants when a sum of exponentials was used to describe macroscopic current decay. For Kv4 channels, the indicated membrane potential was not always the most depolarized tested in these studies because the voltage dependence of the weighted time constant exhibits a J-shape Fineberg et al., 2012. When only a t_0.5_ (time to 50% decay) was reported, the time constant was approximated using a conversion formula (Tau = t_0.5_ /0.693)*.

d *Time constants of recovery from inactivation are reported at strongly hyperpolarized membrane potentials, where backward rate constants of inactivation gating are expected to dominate the kinetics*.

e *Zhi and Covarrubias, personal communication*.

f *Rocha and Covarrubias, personal communication*.

## Phylogeny, structure and inactivation mechanisms of A-type Kv channels

**Figure 2:**
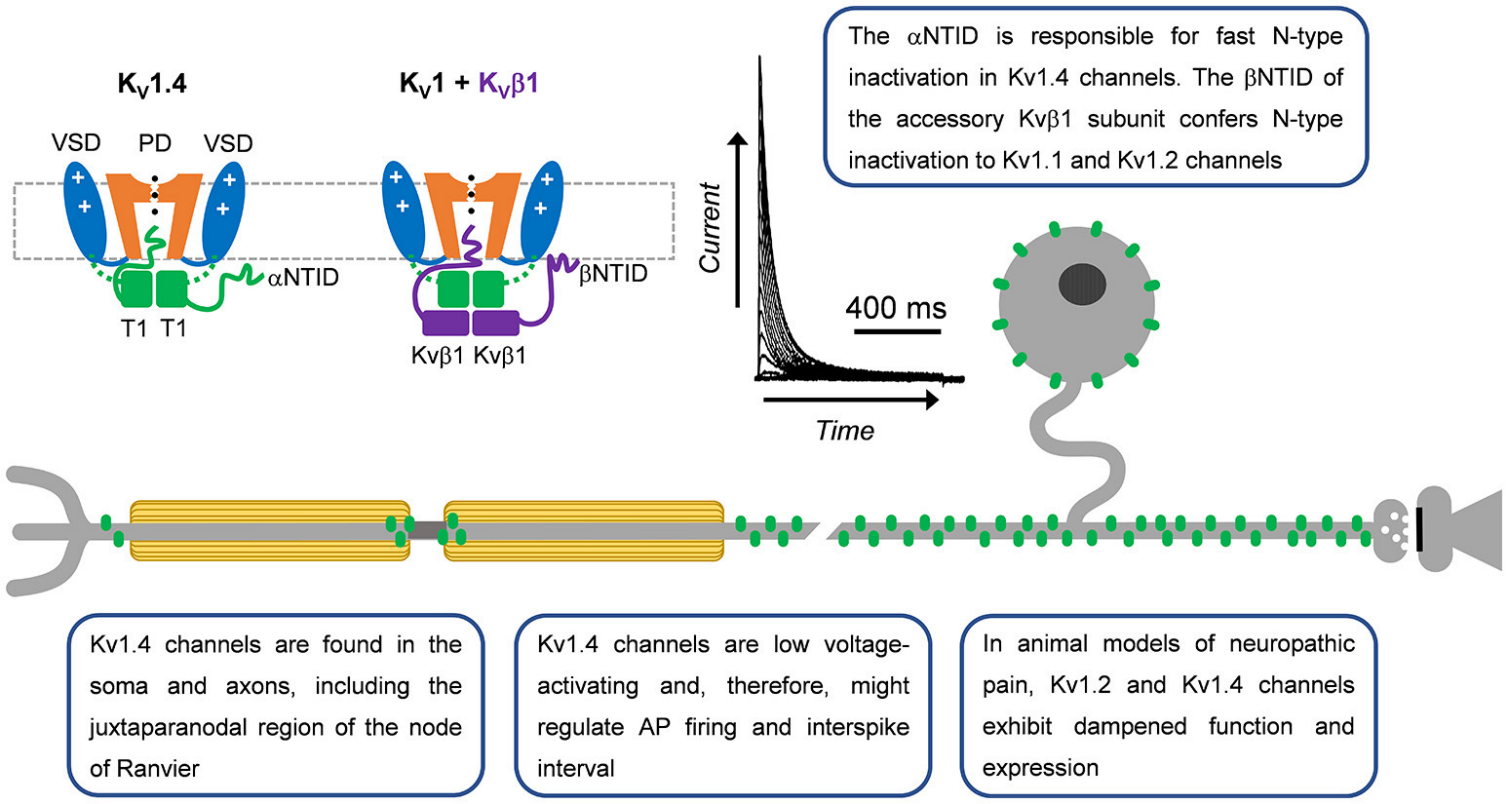
A-type Kv1 channels in primary nociceptive neurons. Cartoon renderings of a Kv1 channel pore-forming a-subunit including characteristic voltage-sensing and pore domains (VSD and PD, respectively) and cytoplasmic tetramerization domain (T1). The alpha subunit N-terminal inactivation domain (αNTID) from one subunit is shown occluding the open pore. The NTID of the Kvβ1 subunit (βNTID) is similarly capable of occluding the open pore. As a result, the current profile exhibits a fast decay over time. A-type Kv1.4 channels are expressed in the somata and axons of unmyelinated and lightly myelinated fibers.

**Figure 3:**
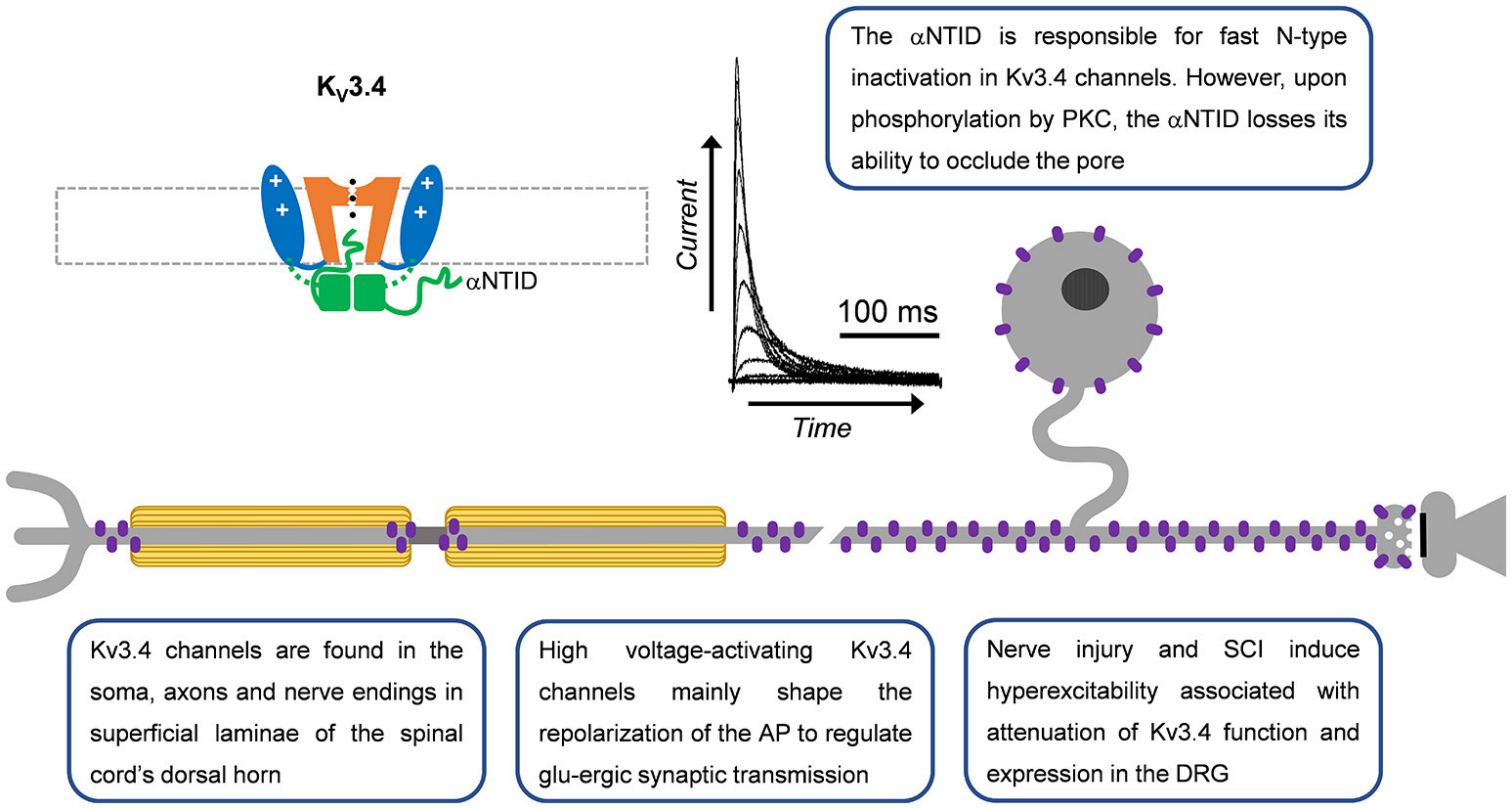
A-type Kv3.4 channels in primary nociceptive neurons. Cartoon rendering of the Kv3.4 channel displaying major functional domains of the pore-forming α-subunit (Figure 2; VSD, PD, T1, and αNTID). The αNTID occludes the open pore to induce a fast inactivating current profile. Kv3.4 immunoreactivity has been detected in somata, axons and nerve endings. In the latter location, attenuation of Kv3.4 function would prolong the AP and, thereby, potentiate glutamatergic (glu-ergic) synaptic transmission, ultimately resulting in increased pain.

**Figure 4:**
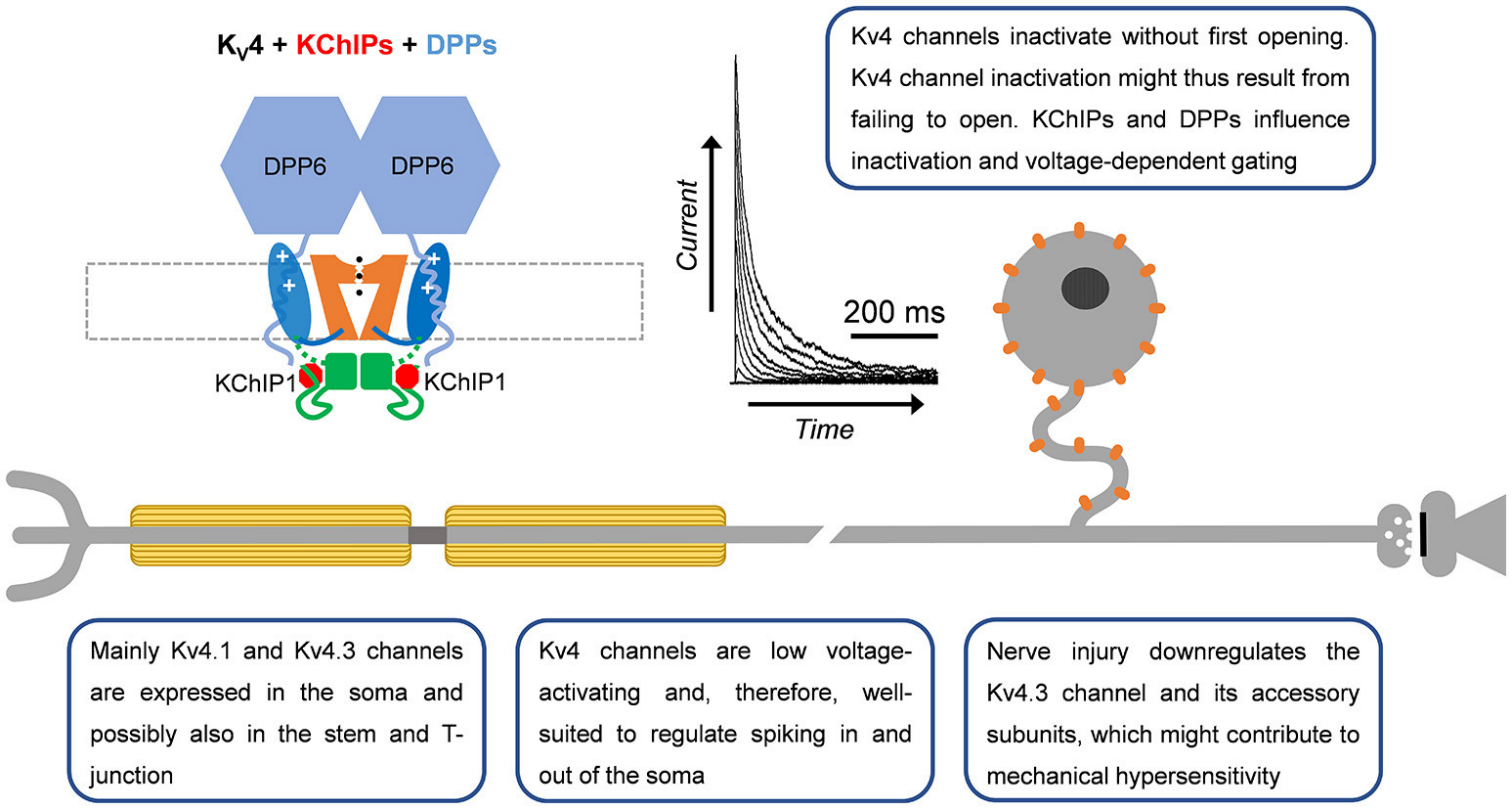
A-type Kv4 channels in primary nociceptive neurons. Cartoon rendering of the Kv4 channel complex including the pore-forming a-subunit with its characteristic Kv channel functional domains (Figure 2; VSD, PD, and T1). Two distinct accessory subunits are also part of this complex: KChIPs and DPPs. Whereas KChIPs are cytoplasmic and bind to the vestigial αNTID to prevent N-type inactivation, DPPs are single-pass membrane spanning proteins that might interact with the VSD to determine the native voltage dependence of Kv4 channels. In addition, the cytoplasmic N-terminal region of DPP6 increases unitary conductance through long-range electrostatic interactions. Kv4.3 immunoreactivity has been detected mainly in somata of small-diameter DRG neurons. Based on the ability of Kv4 channels to regulate backpropagating APs in the CNS, and their particular subcellular localization in DRG neurons, they might act as ‘shock absorbers’ to actively regulate AP propagation into and out of the soma.

In mammals, there are 12 subfamilies of Kv channels (Kv1–Kv12), each with multiple members, that are phylogenetically related to the *Drosophila* Shaker Kv channel. This is in part responsible for the diversity of Kv currents observed in excitable and non-excitable tissues. Whereas Kv1-6, Kv8 and Kv9 channels are closely related to the original Shaker Kv channel, Kv7 and Kv10-12 are more distant relatives. Like all Shaker-related Kv channels, A-type Kv channels are tetrameric assemblies sharing the essential structural features that characterize an individual pore-forming α subunit (from the N-terminus to the C-terminus): the tetramerization T1 domain; six membrane spanning regions including voltage-sensing (S1–S4) and pore domains (S5–S6); and a variable C-terminal domain (Figures 2–4). Despite fundamental similarities that govern voltage dependent gating and K^+^ selectivity, Kv1, Kv3, and Kv4 channels differ in many significant ways (Table 1). Based on the biophysical properties of Kv1.4, Kv3.4, and Kv4s in heterologous and native neuronal systems, the A-type Kv currents can be readily parsed out (Table 1). For instance, while Kv1.4 and Kv4s are low voltage-activating, Kv3.4 is high-voltage activating. It is, however, also possible to distinguish Kv1.4 from Kv4s because the first undergoes slow recovery from inactivation, whereas the latter generally undergo fast recovery from inactivation, even in the absence of auxiliary subunits. Kv3.4 channels also share slow recovery from inactivation, and it is particularly striking that Kv4s exhibit the most hyperpolarized voltage dependence of steady-state inactivation. From a molecular vantage point, Kv1.4, Kv3.4, and Kv4 channels underlie relatively independent Kv current systems because specific structural differences in the T1 domain restrict the formation of heterotetrameric channels to members of the same subfamily (Covarrubias et al., 1991; Li et al., 1992). Distinct mechanisms of inactivation among A-type Kv channels, however, are particularly responsible for the biophysical profile of the corresponding Kv currents.

### Kv1.4 and Kv3.4 channels: N-type inactivating A-type Kv channels

The neuronal Kv1.4 channel generally underlies a low voltage-activating A-type Kv current mainly found in cell bodies and axons (Rasband et al., 2001). It is modestly sensitive to 4-aminopyridine and relatively insensitive to tetraethylammonium (TEA) (Stühmer et al., 1989; Ludewig et al., 1993; Yao and Tseng, 1994; Rasmusson et al., 1995). Fast inactivation of Kv1.4 is determined by the N-terminal inactivation domain (NTID), which operates through a classical “ball-and-chain” mechanism (N-type) similar to that originally identified in the Shaker Kv channel (Murrell-Lagnado and Aldrich, 1993; Tseng-Crank et al., 1993; Baukrowitz and Yellen, 1995; Oliver et al., 1998, 2004; Zhou et al., 2001). Its recovery from inactivation is, however, slow (tens of seconds; Table 1). Additionally, NMR studies have demonstrated that the Kv1.4 channel has two inactivation domains, one that acts as a pore-occluding domain and one that acts as a docking domain (Wissmann et al., 2003). Deletion of either domain slows the rate of inactivation, suggesting that both domains are necessary to promote rapid inactivation in Kv1.4 channels. The Kv1.4 channel interacts with Kvβ subunits related to NADPH reductases, which dock directly below the intracellular T1 domain to modulate surface expression and inactivation gating (Pongs and Schwarz, 2010). In some instances, these subunits confer fast N-type inactivation to other Kv1 channels, such as Kvβ1 when expressed with Kv1.1 or Kv1.2, which on their own are slow inactivating delayed rectifier-type Kv channels (Pongs and Schwarz, 2010). N-type inactivation induced by the Kvβ1 subunit, however, can be negatively modulated by the leucine-rich glioma inactivated gene 1 (Schulte et al., 2006).

The neuronal Kv3.4 channel underlies a high voltage-activating A-type Kv current found in axons and nerve terminals (Rudy et al., 1999; Rudy and McBain, 2001; Brooke et al., 2004; Kaczmarek and Zhang, 2017). It is hypersensitive to 4-aminopyridine and TEA at sub-millimolar concentrations, and fast inactivation of Kv3.4 is determined by an N-type mechanism that uses the channel's NTID. Like Kv1.4, Kv3.4 recovery from inactivation is relatively slow (Rudy et al., 1991; Schröter et al., 1991). However, the NTIDs of Kv1.4 and Kv3.4 share no homology. Most significantly, the Kv3.4 NTID bears several protein kinase C (PKC) phosphorylation sites that are only partially shared with Kv3.3 (Covarrubias et al., 1994; Beck et al., 1998; Kaczmarek and Zhang, 2017). Phosphorylation of these sites causes the Kv3.4 channel to switch from fast inactivating A-type to slow/non-inactivating delayed rectifier-type (Covarrubias et al., 1994; Beck et al., 1998; Antz et al., 1999; Ritter et al., 2012). The Kv3.4 channel interacts with promiscuous KCNE β subunits, which are single membrane spanning proteins that can modulate trafficking and gating (Abbott and Goldstein, 2001; Pongs and Schwarz, 2010; Kanda and Abbott, 2012; Kaczmarek and Zhang, 2017).

### Kv4 channels: non-N-type inactivating A-type Kv channels

The neuronal Kv4.1, Kv4.2, and Kv4.3 channels underlie low-voltage activating A-type Kv currents, mainly expressed in somatodendritic compartments (Birnbaum et al., 2004; Jerng et al., 2004; Shah et al., 2010; Carrasquillo and Nerbonne, 2014). They are only modestly sensitive to 4-aminopyridine and highly insensitive to TEA. However, despite having an NTID-like region, Kv4 channels in their native configuration, which includes accessory β subunits, do not undergo N-type inactivation. Instead, the NTID-like region acts as a binding domain for the Kv4 β subunits known as K^+^-Channel-Interacting-Proteins (KChIPs) (An et al., 2000; Bähring et al., 2001; Pioletti et al., 2006; Wang et al., 2007; Covarrubias et al., 2008; Jerng and Pfaffinger, 2014). Under these conditions, Kv4 channels undergo fast inactivation through a distinct mechanism involving an apparent desensitization to voltage (Bähring and Covarrubias, 2011). Thus, in contrast to N-type inactivation present in Kv1.4 and Kv3.4 in which the channel is inactivated only after opening, closed-state inactivation is the primary pathway of inactivation in neuronal Kv4 channels (Fineberg et al., 2012, 2016). Moreover, unlike Kv1.4 and Kv3.4, Kv4 channels, in their native configuration, exhibit fast recovery from inactivation (Amarillo et al., 2008; Jerng and Pfaffinger, 2014). Additionally, Kv4 channels interact with another class of β subunits known as dipeptidyl peptidase-like proteins (DPPs) that also impact Kv4 properties in native tissues (Nadal et al., 2003; Amarillo et al., 2008; Covarrubias et al., 2008; Maffie and Rudy, 2008; Jerng and Pfaffinger, 2014). These accessory proteins help determine the subthreshold range of membrane potentials over which Kv4 channels typically operate in the brain (Dougherty and Covarrubias, 2006; Dougherty et al., 2008; Maffie and Rudy, 2008). In addition, they are significantly responsible for the fast recovery from inactivation that characterizes Kv4 channels and determine the native unitary conductance (Kaulin et al., 2009). In some instances, the intracellular N-terminus of specialized DPPs can introduce fast N-type inactivation to Kv4 channels, a role that resembles that of the Kvβ1 subunit acting on Kv1 channels (Dougherty and Covarrubias, 2006; Amarillo et al., 2008; Jerng et al., 2009; Kaulin et al., 2009; Nadin and Pfaffinger, 2010).

## A brief history of A-type Kv currents in mammalian DRG neurons and pain

Early patch-clamping studies in acutely dissociated DRG neurons reported low voltage-activating 4-aminopyridine-sensitive A-type Kv currents predominately expressed in small-diameter neurons (Kostyuk et al., 1981; Pearce and Duchen, 1994). Suggesting a physiological role of these currents, exposure to millimolar concentrations of 4-aminopyridine broadened the action potential (AP) in these neurons (Pearce and Duchen, 1994). Gold et al. subsequently described three distinct A-type Kv currents in acutely dissociated rat DRG neurons (Gold et al., 1996). While the high voltage-activating A-type current was predominately found in small, capsaicin positive neurons, subthreshold A-type currents could be detected in small- medium- and large-diameter neurons (Gold et al., 1996). Others verified these observations independently and demonstrated widespread expression of A-type Kv currents in DRG neurons from rat, mouse, rabbit, and guinea pig (Safronov et al., 1996; Everill et al., 1998; Stewart et al., 2003; Phuket and Covarrubias, 2009; Chen et al., 2011; Du and Gamper, 2013).

In multiple chronic pain models, A-type Kv currents in the DRG are reduced. In a spinal nerve ligation model of chronic pain, Everill, and Kocsis first showed reduced A-type Kv currents in Aβ fibers (Everill and Kocsis, 1999). The affected current was sensitive to 4-aminopyridine but not to dendrotoxin, a Kv1.1/1.2/1.6-specific inhibitor (Everill and Kocsis, 1999). These authors hypothesized that decreasing A-type currents in larger sensory neurons (Aβ fibers) may increase firing of primary afferent neurons in the injury model. Additional studies found reduced A-type Kv currents in small-diameter DRG neurons following 2,4,6-trinitrobenzenesulfonic (TNBS) acid induced colitis and pancreatitis as well as models of temporomandibular joint pain, gastric ulcers, and chronic nerve compression (Stewart et al., 2003; Takeda et al., 2006; Xu et al., 2006; Yan et al., 2007; Zhang et al., 2007). Multiple studies reported A-type Kv current reduction possibly resulting from hyperpolarizing shifts in the steady-state inactivation curves, which was associated with increased AP firing (Everill and Kocsis, 1999; Stewart et al., 2003; Takeda et al., 2006). Although dampening of the A-type Kv currents in DRG neurons is linked to persistent pain in multiple chronic pain models, the Kv channels underlying the decrease are only beginning to be identified.

## Function and dysfunction of A-type Kv channel subtypes in DRG neurons

### Kv1.4

Kv1.4 was first identified in cultured DRG neurons via immunohistochemistry (Ishikawa et al., 1999). These studies have shown expression of Kv1.4 in the neuronal soma and axon (Figure 2) (Ishikawa et al., 1999; Rasband et al., 2001). In intact ganglia, Kv1.4 channel immunoreactivity is the only Kv1 channel in small diameter DRG neurons (Rasband et al., 2001) and is the primary Kv1 channel found in isolectin B4 (IB4) positive neurons (Vydyanathan et al., 2005). Kv1.4 immunoreactivity supports electrophysiological recordings showing a Kv1.4-like current in small to medium size DRG neurons (Gold et al., 1996; Safronov et al., 1996; Everill et al., 1998; Vydyanathan et al., 2005). Despite its presence, no specific pharmacological tools are available to probe its role in DRG physiology, forcing researchers to use less specific inhibitors like 4-aminopyridine (Vydyanathan et al., 2005). Subsequent studies confirmed the expression of Kv1.4 channels in the DRG using immunohistochemistry, immunoblotting, and PCR (Yang et al., 2004; Tanimoto et al., 2005; Takeda et al., 2008; Qian et al., 2009; Cao et al., 2010; Duan et al., 2012; Zhu et al., 2012; Li et al., 2014)

A number of signaling processes are capable of modulating Kv1.4 biophysical properties and expression (Figure 2). The fast inactivation kinetics of Kv1.4 are modulated by calcium dependent phosphorylation cascades (Roeper et al., 1997). Ca^2+^/calmodulin dependent protein kinase II (CaMKII) and calcineurin regulate the inactivation profile of Kv1.4. CaMKII phosphorylates S123, an N-terminal residue, which results in slower inactivation kinetics and accelerated recovery from inactivation (Roeper et al., 1997). Conversely, dephosphorylation by calcineurin reverses these effects (Roeper et al., 1997). Both key enzymes are regulated by Ca^2+^, which makes this modulation of Kv1.4 dependent on intracellular changes in Ca^2+^ concentration. Kv1.4 is also regulated by protein kinase A (PKA). Neuronal activity induces PKA-dependent phosphorylation of Kv1.4 Ser229, which reduces macroscopic currents (Tao et al., 2005). Activation of transforming growth factor β1 (TGFβ1) reduces Kv1.4 expression and A-type current density (Zhu et al., 2012). Additionally, a cysteine at position 13 has been shown to be involved in oxidation dependent loss of inactivation (Ruppersberg et al., 1991). Although all of these modulations may occur in neurons, only modulation by TGFβ1 has been shown to occur in DRG neurons (Zhu et al., 2012). Expression of Kv1.4 mRNA is reduced by extracellular UTP through P2Y2 receptors (Li et al., 2014). In heterologous expression systems, the auxiliary subunits KCNE1 and KCNE2 can coassemble with Kv1.4 channels and inhibit trafficking to the cell membrane in a process which can be overcome by heteromultimers consisting of Kv1.1 and Kv1.4 channels (Kanda et al., 2011a,b). In addition, Kvβ2.1 subunits are found in DRG neurons but their impact on Kv1.4 is unknown (Rasband et al., 2001).

Even though Kv1.4 channels were shown immunohistochemically in the DRG, little is known about their role in controlling AP properties in this tissue. Indirect evidence using 4-aminopyridine suggests that Kv1.4 channels in IB4 positive neurons may control AP latency and firing frequency (Vydyanathan et al., 2005). Decreases of Kv1.4-like currents by TGFβ1 activation results in a depolarization of the resting membrane potential, a decrease in rheobase, and broadening of the AP (Zhu et al., 2012). However, the changes following TGFβ1 activation are likely not to result from Kv1.4 channels alone as multiple channels would be affected by 4-aminopyridine.

Converse to the sparse data regarding Kv1.4 channel physiology and modulation in the DRG, expression changes associated with persistent pain have been well documented (Table 2). In diabetic neuropathic pain, mRNA levels of Kv1.4 channels are significantly reduced and there is a reduction in A-type Kv currents in medium to large DRG neurons (Cao et al., 2010). This reduction in Kv1.4 channel mRNA and A-type currents is dependent on brain derived neurotrophic factor (BDNF). Treatment of neurons from diabetic animals with anti-BDNF antibodies restores the currents and Kv1.4 transcripts (Cao et al., 2012). Following an electrical burn injury, the expression level of Kv1.4 channel mRNA and Kv channel current density are reduced (Chen et al., 2011). In bone cancer, Kv1.4 protein expression is up-regulated on post-tumor day 14 with a subsequent decline to control levels (Duan et al., 2012). This upregulation was thought to be due to upregulation in non-injured IB4 positive neurons (Duan et al., 2012). Knockdown of Kv1.4 channels using siRNA induces mechanical allodynia and eliminates the analgesic effects of the compound diclofenac in bone cancer animals (Duan et al., 2012). In axotomy and chronic axon constriction injury models, Kv1.4 channel immunostaining and mRNA levels are decreased substantially in the DRG both ipsilateral and contralateral to injury (Kim et al., 2002; Park et al., 2003; Yang et al., 2004; Li et al., 2014). Kv1.4 channel expression is similarly reduced in ipsilateral DRGs following spinal nerve ligation (Rasband et al., 2001). Following spinal transection, nociceptive bladder sensory neurons show a decrease in A-type Kv current density and a leftward shift in the steady state inactivation curve concurrent with a decrease in mRNA and protein expression of Kv1.4 channels (Takahashi et al., 2013). Other pain models including pancreatitis, inflammatory bowel disease and temporomandibular joint pain have also shown decreases in Kv1.4 expression (Takeda et al., 2008; Zhu et al., 2012; Chen et al., 2013). Interestingly, one study indicated that Kv1.4 may act in a compensatory manner by being upregulated in the juxtaparanodal regions of axons following a sciatic nerve transection (Calvo et al., 2016). After the injury, both Kv1.1 and Kv1.2 become mislocalized and exhibit reduced expression in the juxtaparanodal regions of DRG axons (Calvo et al., 2016). Despite the downregulation of these channels, there is an upregulation of Kv1.4 and Kv1.6 (Calvo et al., 2016). This is interesting considering that Kv1.4 channel upregulation replaces a delayed rectifier with an A-type Kv current. These changes are positively correlated with the proximity to the axonal injury (Calvo et al., 2016). By contrast, two studies on persistent pain states independently showed no change in Kv1.4 channel mRNA, including axotomy (Ishikawa et al., 1999) and irritable bowel syndrome (Qian et al., 2009). Based on electrophysiological studies conducted in heterologous expression systems as well as in DRG neurons, the biophysical properties of the DRG Kv1.4 current appear to most closely resemble the properties of the Kv1.4/Kvβ1.1 complex, suggesting that perhaps the channel exists as a supramolecular complex in DRG neurons (Table 1).

**Table 2:** Pain model-induced changes in A-type Kv channel expression, function and modulation in DRG neurons.

	**Kv1.4**	**Kv3.4**	**Kv4.x**
Sciatic Nerve Ligation	↓IR	↓IR	↓mRNA, ↓IR
Axotomy	↓↔mRNA ↓IR		
Diabetes	↓mRNA, ↓${\text{I}}_{\text{A}}^{1}$	↑mRNA	↓mRNA, ↓I_A_
	↑BDNF		↑PO_4_, ↑BDNF
			↑MAPK
Spinal Cord Injury		↓I_A_, ↓IR^2^	
		↓inactivation rate	
		↔total protein	
		↔mRNA^3^	
		↔CaN, ↑RCAN1	
Bone Cancer	↑IR^4^	↓IR	↑protein
Oxaliplatin induced			↓I_A_,↓protein
Inflammatory Bowel Disease	↓↔mRNA		↓I_A_, ↓ protein
			leftward shifted SSI
			↑PO_4_, ↑MAPK
Spinal Cord Transection	↓mRNA, ↓I_A_ ↓protein		
	leftward shifted SSI		
Temporomandibular Joint	↓IR		
Electrical Burn	↓mRNA, ↓I_A_		
Chronic Constriction	↓mRNA, ↓IR		↓mRNA
Sciatic Nerve transection	↑IR^5^		
Pancreatitis	↓mRNA		
	↑TGFβ1		
Vibration induced			↓mRNA

*↑ with green text indicates an increase, ↓ with red text indicates decrease, ↔ with black text indicates no change, and combined arrows (↑↓ ↔) indicates conflicting evidence. Abbreviations corresponding to particular molecular and functional changes are indicated as follows: IR, immunoreactivity, I_A_, current, PO_4_, phosphorylation, SSI, steady state inactivation. Evaluation of protein expression is generally based on western blot analysis. All studies are cited in the text. Some experiments were done in specific settings as noted: ^1^in medium- to large-diameter DRG neurons, ^2^cell surface IR, ^3^single cell mRNA, ^4^in uninjured IB4+ neurons, ^5^in juxtaparanodal regions*.

### Kv3.4

Kv3.4 immunoreactivity in the superficial rat spinal dorsal horn provided strong evidence for the expression of Kv3.4 channels in DRG nociceptors (Brooke et al., 2004; Chien et al., 2007; Muqeem et al., 2018). Specifically, the axon, soma and presynaptic terminals of rat DRG neurons demonstrated significant Kv3.4 immunoreactivity (Figure 3; Chien et al., 2007; Ritter et al., 2015a; Zemel et al., 2017; Muqeem et al., 2018). In the somata of DRG neurons, Kv3.4 channels are found in all neurons, although it appears to be especially enriched in small-diameter neurons (Chien et al., 2007; Ritter et al., 2012, 2015a; Zemel et al., 2017). The immunoreactivities of Kv3.4, Nav1.8, and TRPV1 colocalize, which is consistent with expression in nociceptors (Chien et al., 2007; Ritter et al., 2015a). Kv3.4 currents were first identified from 7 day old rat pups using the cell-attached patch-clamp method and a depolarized conditioning pulse (−30 mV) to inactivate low voltage-activating A-type Kv channels (Ritter et al., 2012). These currents closely resemble those induced by heterologously expressed Kv3.4 channels (Covarrubias et al., 1994; Beck et al., 1998), are hypersensitive to TEA, and are knocked-down with Kv3.4 siRNA (Ritter et al., 2012; Table 1). Retrospective analysis of whole-cell currents recorded by Gold et al. in the DRG demonstrated that the I_aht_ current (named for A-type, high-threshold) also had Kv3.4-like properties (Gold et al., 1996). The majority of neurons that exhibited I_aht_ were also capsaicin responsive. This suggests that Kv3.4 currents are expressed in putative nociceptors. Kv3.4 currents with similar properties have since been additionally isolated in DRG neurons from adult male and female rats, suggesting that Kv3.4 expression is stable during postnatal development (Ritter et al., 2015a,b). Kv3.3 mRNA and immunoreactivity are also found in larger neurons of the DRG but are not highly expressed or as of yet implicated in persistent pain syndromes (Bocksteins and Snyders, 2012; Ritter et al., 2012).

Kv3.4 channel function is modulated by oxidation, phosphorylation and ancillary proteins (Figure 3; Ruppersberg et al., 1991; Covarrubias et al., 1994; Baranauskas et al., 2003; Kanda et al., 2011a). Inactivation of Kv3.4 channels is slowed by oxidation and phosphorylation of the NTID in heterologous systems (Ruppersberg et al., 1991; Covarrubias et al., 1994). Cysteine oxidation at position 6 removes inactivation by forming a disulfide bond between the NTID and another part of the channel (Ruppersberg et al., 1991). Phosphorylation of the Kv3.4 channel NTID at S8, S9, S15, and S21 by PKC alters the structure of the NTID thereby slowing inactivation (Covarrubias et al., 1994; Beck et al., 1998; Antz et al., 1999; Ritter et al., 2012). This PKC mediated action may occur through several receptors, including metabotropic glutamate receptors and serotonin receptors (Velasco et al., 1997; Kruse et al., 2012). In DRG neurons, the modulation by G-protein coupled receptors occurs by a membrane-delimited mechanism suggesting the presence of a Kv3.4 channel-receptor-PKC complex (Ritter et al., 2012). The phosphatase calcineurin (CaN) seemingly opposes the activity of PKC as inhibiting CaN with small molecules or overexpressing the regulator of calcineurin 1 (RCAN1) reduces Kv3.4 inactivation (Zemel et al., 2017). High levels of phosphatidylinositol 4,5-bisphosphate (PIP2) may also alter Kv3.4 channel inactivation but this has not been confirmed in a physiological setting (Oliver et al., 2004; Kruse et al., 2012). Kv3.4 channels are also modified by KCNE (formerly MiRP) proteins in heterologous expression systems and natively in skeletal myocytes (Abbott et al., 2001; Abbott and Goldstein, 2002; Kanda et al., 2011a; Kanda and Abbott, 2012). In these cells, KCNE proteins modulate time and voltage-dependent properties and trafficking of the Kv3.4 channel. The inhibition of trafficking by KCNE proteins may be overcome by formation of heteromultimers with Kv3.1 channels (Kanda et al., 2011b). The modulation of Kv3.4 channels by KCNE has yet to be demonstrated in neurons. Kv3.4 mRNA decreases in response to extracellular UTP, a response that is downstream to the G-protein coupled P2Y2 receptors (Li et al., 2014). There are also three known Kv3.4 mRNA splice variants with unknown specific roles (Rudy et al., 1999; Rudy and McBain, 2001). Female rat nociceptors express all three variants, although the Kv3.4b transcript is expressed at very low levels (Ritter et al., 2015a).

Our work and that of others have shown that Kv3.4 channels are optimized to regulate repolarization of the nociceptor AP and thus its duration (Ritter et al., 2012, 2015b; Liu et al., 2017). Consistent with this role, knock-down or inhibition of Kv3.4 in the DRG broadens the AP and phosphorylation of the channel shortens the AP (Ritter et al., 2012, 2015a; Muqeem et al., 2018). AP clamp techniques reveal that Kv3 currents provide a large proportion of the repolarizing charge during the nociceptor AP (Ritter et al., 2015b; Liu et al., 2017). Additionally, pharmacological inhibition of Kv3.4 channels in the DRG was found to potentiate excitatory post-synaptic currents in superficial layers of the dorsal horn (Muqeem et al., 2018). This finding suggests that modulation of Kv3.4 currents or channels presynaptically in the DRG may impact synaptic transmission in the nociceptive pathway. Kv3.4 channels might also influence spiking in nociceptors. In dynamic-clamp experiments, the addition of computer-generated Kv3.4 currents decreases repetitive firing (Ritter et al., 2015a). This could be due to a significant open probability of the Kv3.4 channel near the threshold of the AP (Ritter et al., 2012, 2015a) or due to reopening of Kv3.4 channels during recovery from inactivation induced by hyperpolarization (Ruppersberg et al., 1991).

Several chronic pain models exhibit dysfunction in Kv3.4 channels (Table 2). In a sciatic nerve ligation model, Kv3.4 immunoreactivity is reduced in DRG nociceptors (Chien et al., 2007). Following implantation of a bone tumor near the sciatic nerve, immunoreactivity of DRG Kv3.4 channels was reduced as determined by western blot (Duan et al., 2012). Finally, in a model of unilateral spinal cord contusion, Kv3.4 current amplitude, inactivation, and channel membrane expression are reduced in the DRG (Ritter et al., 2015a; Zemel et al., 2017). Western blot and single-cell quantitative PCR results indicate that total protein and mRNA in the DRG had not changed, suggesting a possible post-translational effect (Ritter et al., 2015a). We proposed that spinal cord injury might induce DRG Kv3.4 channel dysfunction through alteration of its phosphorylation state. Although PKC was known to phosphorylate Kv3.4 channels causing an acute loss of inactivation, nothing was known about the phosphatases that countered PKC activity. We found that pharmacological inhibition of CaN was sufficient to not only slow inactivation, but also attenuate Kv3.4 currents (Zemel et al., 2017). These modulations are dependent on the presence of the NTID PKC sites at S8, S9, S15, and S21 (Zemel et al., 2017). Subsequently, we found that the regulator of CaN, RCAN1, is upregulated in DRG neurons following spinal cord injury leading to inhibition of CaN, causing slowing of Kv3.4 channel inactivation, attenuation of Kv3.4 currents, and slowing of the nociceptor action potential repolarization (Zemel et al., 2017). These studies strongly suggest that a decrease in Kv3.4 activity has a substantial effect on nociceptor excitability after injury. Intrathecal injection of antisense Kv3.4 oligonucleotides induces mechanical hypersensitivity in rats, which is consistent with the role for Kv3.4 channels in nociception and the development of chronic pain (Chien et al., 2007). Converse to all other Kv3.4 channel studies in pain models, in a diabetic neuropathy model, Kv3.4 mRNA increased in the entire ganglia (Cao et al., 2010). The biophysical properties of Kv3.4 channels expressed in heterologous expression systems almost exactly mirror those of the Kv3.4 current isolated from DRG neurons, indicating a homomultimeric neuronal configuration (Table 1).

### Kv4.1, Kv4.2, and Kv4.3

While Kv4.x channel mRNA was first reported in whole-ganglia isolates (Kim et al., 2002; Park et al., 2003; Winkelman et al., 2005), later work would verify expression of Kv4 channels in predominately the somata of small and large diameter nociceptors and the dorsal horn of the spinal cord via immunohistochemistry (Huang et al., 2005; Hu et al., 2006; Chien et al., 2007). These findings were consistent with previously identified low voltage-activating A-type Kv currents in DRG neurons (Gold et al., 1996). Through the use of a specific dominant negative construct and selective neurotoxins (heteropodatoxin and phirxotoxin), along with single cell RT-PCR, Kv4 channels have been established as the molecular correlates of subthreshold A-type currents in DRG neurons (Sculptoreanu and de Groat, 2007; Phuket and Covarrubias, 2009; Sculptoreanu et al., 2009; Yunoki et al., 2014). Although all three Kv4 mRNA isoforms (Kv4.1-4.3) are expressed in whole DRG preparations (Kim et al., 2002; Winkelman et al., 2005), there is evidence for differential expression. Kv4.1 mRNA is expressed in DRG neurons of all sizes, Kv4.2 mRNA is absent from small-diameter DRG neurons and Kv4.3 mRNA is mainly found in small-diameter DRG neurons (Phuket and Covarrubias, 2009; Matsuyoshi et al., 2012; Yunoki et al., 2014). Immunohistochemistry supports the predominant expression of Kv4.3 over Kv4.2 channels in the DRG (Huang et al., 2005; Hu et al., 2006; Phuket and Covarrubias, 2009). Kv4.3 channels are found predominantly in IB4+ neurons and are co-expressed with the nociceptor markers Nav1.8 and TRPV1, but not CGRP (Huang et al., 2005; Chien et al., 2007; Phuket and Covarrubias, 2009; Duan et al., 2012; Yunoki et al., 2014).

Kv4 channel expression and function in the DRG is modulated by several signaling pathways and accessory subunits. Gene expression is regulated by the neuron restrictor silencer factor (REST), which binds to the promoter of Kv4.3 and recruits histone deacetylase 4 (HDAC4) to repress transcription of the Kv4.3 gene (Ballas and Mandel, 2005; Uchida et al., 2010). Interestingly, REST expression has been shown to be increased in a partial sciatic nerve ligation model of nerve injury (Rose et al., 2011). Expression of Kv4.2 and Kv4.3 in whole DRG tissue is reduced by the application of brain-derived neurotrophic factor (BDNF) and neurotrophin 3 (NT-3), two factors upregulated in chronic pain states (Park et al., 2003). Blocking BDNF function, or the function of tyrosine kinases downstream of BDNF, increases Kv4.2 and Kv4.3 mRNA and the A-type current in DRG neurons (Cao et al., 2010). Extracellular UTP decreases A-type Kv currents along with Kv4.2 expression in the trigeminal ganglion via the P2Y2 receptor (Li et al., 2014). Kv4 channels are also regulated by phosphorylation (Jerng et al., 2004; Kim and Hoffman, 2008). Phosphorylation of threonine 602 in Kv4.2 by mitogen-activated protein kinases (MAPK) causes attenuation of the low voltage-activating A-type Kv current in DRG neurons (Grabauskas et al., 2011). In addition to signaling pathways, Kv4.x channels are modulated by auxiliary subunits. DPP10, KChIP1, KChIP2, and KChIP3 are expressed in the DRG (Phuket and Covarrubias, 2009; Cheng et al., 2016; Kuo et al., 2017). Recently the discovery of a Kv4.3/KChIP1/KChIP2/DPP10 complex was found in DRG neurons via co-immunoprecipitation studies (Kuo et al., 2017). Knockdown of any component of the Kv4 channel complex reduces the expression of the other components and increases excitability of IB4+ nociceptors (Kuo et al., 2017). Components of this complex were found to be downregulated in a spinal nerve ligation (SNL) model of chronic pain (Kuo et al., 2017). Overexpressing various components of this complex rescued downregulated Kv4.3 currents as well as attenuated DRG excitability and pain phenotypes of injured animals.

To date, several studies have implicated Kv4 channel dysfunction in chronic pain (Table 2). In both chronic constriction of the sciatic nerve and axotomy, expression of Kv4.2, and Kv4.3 mRNA is reduced in DRG neurons (Kim et al., 2002; Park et al., 2003; Furuta et al., 2012). Subsequent studies showed that Kv4.3 DRG immunoreactivity is reduced by 40% following nerve ligation (Chien et al., 2007). Vibration induced injury decreases Kv4.3 mRNA in the nerves innervating the affected side (Conner et al., 2016). In streptozotocin (STZ)-induced diabetic neuropathy, there are robust decreases in A-type Kv currents and Kv4 expression in putative nociceptors after disease onset (Cao et al., 2010; Grabauskas et al., 2011; Sun et al., 2011). STZ-induced diabetes causes a ~50% BDNF-dependent reduction in the expression of Kv4.2 and Kv4.3 mRNAs in DRG neurons (Cao et al., 2010). Following STZ treatment, Kv4.2 channels are phosphorylated by MAPK with a corresponding decrease in the A-type Kv currents (Grabauskas et al., 2011). The use of MAPK inhibitors restores both the A-type Kv current in nociceptors as well as reduces the anorectal sensitivity induced by STZ (Grabauskas et al., 2011). Increased MAPK-dependent phosphorylation of Kv4.2 followed by attenuation of the A-type current is also seen in a model of irritable bowel syndrome induced by butyrate (Xu et al., 2012). In a second model of colonic hypersensitivity, there is downregulation of Kv4.3 protein as well as a leftward shift in the voltage-dependence of inactivation in IB4+ DRG neurons (Qian et al., 2009). In both colonic hypersensitivity studies, the decrease in A-type Kv current coincided with a depolarized membrane potential and increased excitability, both of which are predicted by a loss of Kv4 channels (Qian et al., 2009; Xu et al., 2012). Kv4.3 protein and currents are also downregulated in a model of chemotherapy induced neuropathy resulting from oxiliplatin administration (Viatchenko-Karpinski et al., 2018). Currently only one model has shown an increase in Kv4 channels following injury. In bone cancer pain, Kv4.3 immunoreactivity is increased in the weeks following cancer development which the authors hypothesize is a protective mechanism to dampen excitability (Duan et al., 2012).

More directly implicating Kv4 channels in the development of chronic pain, knockdown of Kv4.3 channels induces hypersensitivity. Three days of intrathecal administration of Kv4.3 antisense oligonucleotides induces mechanical allodynia but not thermal hyperalgesia (Chien et al., 2007). A separate group also injected Kv4.3 channel antisense oligonucleotides and have shown increased sensitization to vibration (Conner et al., 2016). In a bone cancer model of chronic pain, injection of Kv4.3 siRNA in the lumbar spinal cord prohibits the ability of diclofenac to reverse the mechanical allodynia phenotype with no effect on thermal hyperalgesia (Duan et al., 2012). These three studies present a strong case for the involvement of Kv4.3 channels in mechanical allodynia phenotypes. Previous studies have extensively characterized the biophysical properties of Kv4 channels with various β subunits in heterologous expression systems as well as the native configuration present in DRG neurons; these studies suggest that the DRG Kv4 channels likely exist as heteromultimers and in ternary complexes (Table 1).

## A-type Kv channels as therapeutic targets in chronic pain

If the decrease in A-type currents contributes to chronic pain, restoring the currents should significantly attenuate the chronic pain phenotype. The nonsteroidal anti-inflammatory drug, diclofenac, increases A-type Kv currents, and reverses the pain phenotype in a bone cancer model of chronic pain (Duan et al., 2012). It should be noted, however, that diclofenac interacts with multiple Kv channels and will likely impact other currents within the pain pathway (Huang et al., 2013). Regardless, other experiments have shown that using drugs to increase A-type Kv currents or synthetically increasing A-type currents reverses excitability changes in the DRG (Sculptoreanu et al., 2004; Ritter et al., 2015a). To target A-type Kv channels, three possible approaches might be considered. First, specific pharmaceuticals that might act as A-type channel “openers” by altering biophysical properties. These compounds might induce (1) a hyperpolarizing shift in the voltage dependence of activation, (2) a depolarizing shift in the voltage dependence of inactivation; and/or (3) an increase in the maximum open probability. For instance, novel positive modulators acting selectively on certain Kv3 channels by stabilizing their open state (Brown et al., 2016). By shifting the voltage dependence of activation, it should function to dampen excitability as shown in dynamic clamp experiments (Ritter et al., 2015a). Second, there are pharmacological and biological manipulations that upregulate the expression of Kv channels and/or β subunits that promote Kv channel trafficking, surface expression and/or conductance, such as Kvβ1-3, KCNE, DPP, and KChIP (Amarillo et al., 2008; Kaulin et al., 2009; Pongs and Schwarz, 2010; Kanda et al., 2011a,b; Sun et al., 2011; Kuo et al., 2017). These manipulations might utilize virus-based transduction to directly upregulate K^+^ channel components in the DRG (Xu et al., 2003; Zheng et al., 2009; Ma et al., 2010; Yu et al., 2011; Kuo et al., 2017), or pharmacologic and genetic tools that modulate transcription factor activity (REST, BDNF, and estrogen) to promote A-type Kv channel expression (Vullhorst et al., 2001; Cao et al., 2010; Uchida et al., 2010; Wang et al., 2010). Finally, targeting signaling pathways that converge on A-type channels may revert or prevent the development of chronic pain. A recent study found that upregulation of a micro-RNA cluster (mir-17-92) after L5 spinal nerve ligation or its experimental overexpression reduced the expression of all three A-type Kv channels expressed in DRG (Sakai et al., 2017). This finding provides a possible explanation for the loss of multiple A-type channels that result from the same insult to peripheral and central tissues. Targeting this micro-RNA cluster after injury with antisense oligomers could prove therapeutic, although more studies are necessary to pursue testing of this approach. Regarding specific channels, two significant cases come to mind. PKC-dependent phosphorylation and CaN-dependent dephosphorylation of Kv3.4 channels underlie a tight regulation of the channel that may be manipulated to alter the firing of nociceptors (Ritter et al., 2012, 2015a; Zemel et al., 2017). For instance, activation of PKC would result in phosphorylation of well-defined serine residues on the N-terminus of Kv3.4, which would then result in loss of N-type inactivation and an overall strengthening of the current response; this would lead to a shortening of the DRG action potential (Ritter et al., 2012) and likely overall dampened pain transduction. Kv1.4 channel phosphorylation via PKA and Ca^++^/calmodulin dependent kinase will increase expression and slow inactivation (Roeper et al., 1997; Tao et al., 2005) which might inhibit firing by increasing the threshold for firing.

## Conclusions and perspective

DRG neurons express a variety of A-type Kv channels that regulate membrane excitability. The currents mediated by these ion channels are reduced in multiple persistent pain models (Table 2), which might contribute to neuronal hyperexcitabilty and the resulting persistent pain state. In support of this idea, selective knockdown of A-type Kv channels induces pain phenotypes and procedures that re-express A-type currents show beneficial effects (Chien et al., 2007; Duan et al., 2012; Ritter et al., 2015a; Conner et al., 2016). Future DRG work on the molecular and physiological properties of A-type Kv channel subtypes and the signaling pathways that regulate them would help gain a better understanding of how chronic pain develops and potentially how it can be rectified.

Despite having knowledge on the identity of specific Kv channels underlying the A-type current in DRG neurons, numerous studies still only report changes in the “A-type current.” Currently Kv3.4 and Kv4 currents can be isolated via electrophysiological methods, molecular probes and toxins (Sculptoreanu and de Groat, 2007; Phuket and Covarrubias, 2009; Ritter et al., 2012; Yunoki et al., 2014). By contrast, Kv1.4 currents have yet to be exclusively identified in the DRG. However, the protocol used by Gold et al. may provide such an isolation method (Gold et al., 1996). Use of siRNA (Duan et al., 2012), knockout animals, or a Kv1.4 channel specific toxin would help elucidate the role of Kv1.4 in DRG neuron physiology.

Molecular biology and biochemistry also play an important role in elucidating components of an A-type current in specific DRG neuron populations. Due to the heterogeneity of cell types within the DRG, analysis of whole DRG tissue lysates is not sufficient to identify changes in channel expression in distinct cell populations. A multipronged approach that includes patch-clamping electrophysiology, single-cell RT-PCR, and immunohistochemistry coupled with high resolution imaging would be necessary to examine ion channel expression in specific DRG neuron subtypes (Phuket and Covarrubias, 2009; Ho and O'leary, 2011; Ritter et al., 2012, 2015a).

In addition to understanding cell-type specific expression, it is important to note that each compartment of the DRG neuron (soma, t-stem, axon, peripheral terminal, and spinal dorsal horn terminal) has different properties and functions. For example, low voltage-activating A-type Kv channels at the T-junction might act as “gate keepers” regulating AP propagation into the soma (Lüscher et al., 1994), and high voltage-activating A-type Kv channels in the spinal dorsal horn nerve terminal might affect neurotransmitter release via regulation of AP repolarization. Kv1.4 channels are found in the soma and axon (Rasband et al., 2001), Kv3.4 channels are expressed throughout all parts of the neuron (Brooke et al., 2004; Chien et al., 2007; Ritter et al., 2015a; Muqeem et al., 2018), and Kv4 channels appear to be restricted to the soma (Chien et al., 2007; Phuket and Covarrubias, 2009). However, most studies only examine changes in the soma. With the advent of new electrophysiological, genetic, and optical techniques and preparations, examining properties of locations outside the soma is becoming possible (Pinto et al., 2008; Szűcs et al., 2009; Johannssen and Helmchen, 2010; Vrontou et al., 2013; Chen et al., 2014; Cui et al., 2016; Hachisuka et al., 2016; Kim et al., 2016; Chisolm et al., 2018).

In different DRG compartments, depending on their voltage- and time-dependent properties and their modulation by second messengers and accessory proteins, A-type Kv channels could regulate membrane potential, spike latency, spike train properties and the AP waveform. Although major advances have been made to understand these roles in central neurons, much less can be said about DRG neurons, which are very heterogeneous and have highly specialized morphology and physiological properties (Granados-Fuentes et al., 2012; Kim and Hoffman, 2012; Rowan et al., 2016; Rowan and Christie, 2017). More progress in this area would hasten elucidation of the relationship between disease-induced alterations in A-type Kv channel expression and function to specific physiological properties of the DRG neuron. Furthermore, this knowledge would enable more concrete understanding of how dysfunction of specific A-type Kv channels leads to pain disorders.

**Figure 5:**
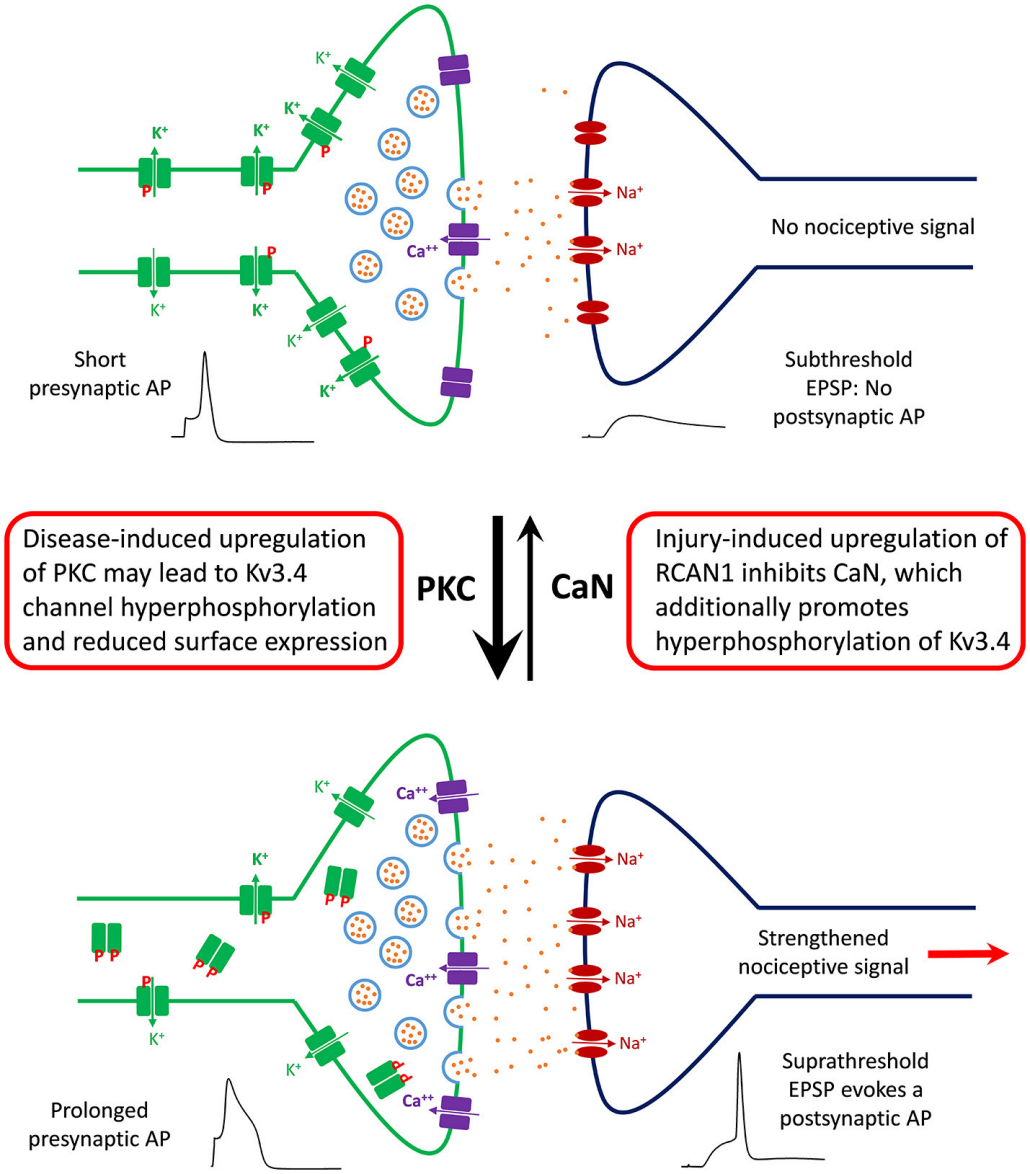
Working model of the role of DRG Kv3.4 channels in nociception and neuropathic pain at the level of the first synapse in the dorsal horn. Under homeostatic conditions, Kv3.4 channels (green blocks) in DRG neurons might play an antinociceptive role by limiting excitatory neurotransmission in the superficial dorsal horn. Kv3.4 channels keep presynaptic excitability in check by regulating AP duration and thereby limiting vesicular Ca^++^-dependent glutamate release. Phosphorylated Kv3.4 channels have an enhanced ability to play this role because they exhibit reduced N-type inactivation. However, following an injury (e.g., SCI and SNL), various factors (inflammation, maladaptive cellular changes, serotonin spillover, etc.) might lead to hyperactivation/upregulation of PKC and inhibition of CaN. Thus, Kv3.4 channels may become hyperphosphorylated, which reduces its presence on the cell membrane of DRG neurons. Consequently, the presynaptic AP is broader, vesicular glutamate release is increased, and a potentiated EPSP crosses threshold to evoke a nociceptive postsynaptic AP that relays a painful signal to the brain. Under chronic conditions, this scenario could underlie a state of persistent neuropathic pain.

Based on our recent work, we have generated a working model that helps explain the function, modulation, and dysfunction of the Kv3.4 channel in DRG nociceptors (Ritter et al., 2012, 2015a,b; Zemel et al., 2017; Muqeem et al., 2018). We propose that a major role of the presynaptic Kv3.4 channel in these neurons is to regulate Ca^2+^-dependent glutamatergic neurotransmission through its ability to regulate the repolarization of the AP that invades the C-fiber nerve terminals (Figure 5). This property depends on the modulation of the Kv3.4 channel's NTID by PKC and CaN. The Kv3.4 NTID hosts four PKC sites per subunit in the Kv3.4 tetramer (S8, S9, S15, and S21). Thus, under normal conditions, nociception is kept in check by maintaining the Kv3.4 NTID modestly phosphorylated mainly at two positions (e.g., S8 and/or S9), which greatly potentiates Kv3.4 activity by reducing its N-type inactivation and, thereby, ensuring rapid AP repolarization. Therefore, Ca^2+^-dependent vesicular release of glutamate and the transmission of the nociceptive signal are limited. However, following neural injury and the resulting inflammatory responses affecting the DRG and the spinal dorsal horn, RCAN1 is upregulated, CaN is, consequently, inhibited and the remaining Kv3.4 NTID sites (S15 and S21) become additionally phosphorylated. Prolonged hyperphosphorylation of the Kv3.4 NTID might then attenuate the Kv3.4 current and eventually lead to reduced surface expression in DRG neurons. The persistent negative modulation of Kv3.4 prolongs the presynaptic AP, leading to increased glutamatergic neurotransmission in the spinal dorsal horn and persistently enhanced nociception. This is a peripheral mechanism that might underlie chronic SCI-induced pain sensitization and other neuropathic pain disorders.

Advancing understanding of the diversity, function and dysfunction of A-type Kv channels in DRG neurons would pave the way to discover more effective methods to treat intractable pain disorders. Currently, a couple of drugs have been shown to upregulate A-type Kv currents but the mechanisms of action are unknown (Li et al., 2010; Duan et al., 2012). The use of gene therapy may also become an important tool in the future (Tsantoulas and McMahon, 2014). Moreover, genetic mouse models and small molecules that specifically target different A-type Kv channel subtypes would help investigate their specific roles in pain under physiological and pathological conditions.

## Author contributions

DR, BZ, and TM drafted, revised and approved of the final manuscript. MC revised and approved of the final manuscript.

### Conflict of interest statement

The authors declare that the research was conducted in the absence of any commercial or financial relationships that could be construed as a potential conflict of interest.
